# Elements of Histology

**Published:** 1899-11

**Authors:** 


Elements of Histology. By E. Klein, M. D., F. R. S., Lecturer on General Anatomy and Physiology, and J. S. Edkins, M. A., M. B., Joint Lecturer and Demonstrator of Physiology in the Medical School of St. Bartholomew's Hospital, London. With 296 illustrations, revised and enlarged edition. Lea Brothers & Co., Philadelphia and New York. 1899.

The last edition of this book was published in 1889, ten years ago, since which time so many additions to the knowledge of minute structural anatomy have been made, that it became necessary to get out another edition with revision and enlargement.

Progress has been made mostly in the knowledge of the structure and life of the cell and nucleus, and especially in the central nervous system and sense organs. The chapters dealing with these subjects have been rewritten. Dr. Edkins has been associated in this edition as one of the joint authors, the chapters relating to the brain, medulla and alimentary canal having been contributed by him.

The size of this book, 4½x6¾ inches, makes it one of the most convenient text-books extant. The evidence of master hands is shown on every page. Nothing better could be said of the book.

